# Platelet-derived sphingosine 1-phosphate induces migration of Jurkat T cells

**DOI:** 10.1186/1476-511X-13-150

**Published:** 2014-09-25

**Authors:** Junko Iino, Makoto Osada, Makoto Kurano, Makoto Kaneko, Ryunosuke Ohkawa, Yumiko Satoh, Shigeo Okubo, Yukio Ozaki, Minoru Tozuka, Nelson H Tsuno, Yutaka Yatomi

**Affiliations:** Department of Clinical Laboratory, The University of Tokyo Hospital, Tokyo, Japan; Department of Clinical Laboratory Medicine, Graduate School of Medicine, The University of Tokyo, Tokyo, Japan; Analytical Laboratory Chemistry, Graduate School of Health Care Sciences, Tokyo Medical and Dental University, Tokyo, Japan; Department of Clinical and Laboratory Medicine, Faculty of Medicine, University of Yamanashi, Yamanashi, Japan; Department of Transfusion Medicine, The University of Tokyo, Tokyo, Japan

**Keywords:** Sphingosine 1-phosphate, Jurkat T-cells, Lymphocytes, Platelets, Migration

## Abstract

**Background:**

The migration of T cell to atherosclerotic lesions is proposed to be involved in the pathogenesis of the atherosclerosis. Sphingosine 1-phosphate (S1P), a bioactive lysophospholipid released from activated platelets, exerts a variety of responses such as cell migration and proliferation, and reportedly induces T cell migration. Accordingly, platelet-T cell interactions may exist based on T cell responses triggered by platelet-derived S1P.

**Methods:**

S1P was measured using two-step lipid extraction followed by high-performance liquid chromatography (HPLC) separation while other phospholipids were determined by an enzymatic assay. The expression of S1P and lysophosphatidic acid receptors on Jurkat T cells was examined by RT-PCR and flow cytometry. Jurkat cell migration by S1P and the supernatant of activated platelets (SAP) was evaluated by a modified Boyden’s chamber assay.

**Results:**

S1P_1_ receptor was confirmed to be expressed on Jurkat T cell by RT-PCR and flow cytometry. S1P at 10-100 nM induced strong Jurkat cell migration, which was inhibited by the S1P_1_ (and S1P_3_) antagonist VPC23019 and the Gi inactivator pertussis toxin (PTX). We found that the supernatant (releasate) of human platelets activated by collagen stimulation, which contains S1P abundantly, induced Jurkat cell migration and that the migration was inhibited by VPC23019 and PTX. In addition, human serum, into which platelet contents (including S1P) are fully released, induced the Jurkat cell migration, which was also inhibited by VPC23019.

**Conclusions:**

Our findings suggest that platelet-derived S1P induces Jurkat T cell migration possibly via S1P_1_. S1P may be a key molecule involved in the responses triggered by platelet-T cell interactions, including atherosclerosis.

## Introduction

Lysophospholipids, such as sphingosine 1-phosphate (S1P), lysophosphatidic acid (LPA), lysophosphatidylserine (LPS), lysophosphatidylinositol (LPI), lysophosphatidylethanolamine (LPE), are important lipid mediators involved in various physiological and pathophysiological events. Among these lipid mediators, much research has been conducted with LPA and S1P, and their important role at an individual level has been confirmed. S1P not only serves as an intermediate metabolite linking sphingolipids and glycerophospholipids, but also exerts strong biological responses, through the S1P receptors expressed on a variety of cell types, including lymphocytes and endothelial cells [1]. More recently, S1P has been shown to facilitate the migration of lymphocytes, particularly T lymphocytes, and S1P signaling has been therapeutically targeted in the treatment of remitting, relapsing multiple sclerosis. Indeed, the sphingosine analogue Fingolimod, also known as FTY720, has been approved by the US Food and Drug Administration for the treatment of multiple sclerosis [2, 3]. FTY720 is phosphorylated by sphingosine kinase 2 to FTY720-P, which markedly reduces the number of circulating lymphocytes in peripheral blood, through downregulation of S1P_1_. In this way, S1P has been focused as a new therapeutic target, and new clinical applications are expected to be available by the development of receptor specific agonist/antagonist or inhibitors of the enzyme involved in S1P metabolism.

The S1P present in the circulation is believed to be mainly derived from erythrocytes, platelets, and vascular endothelial cell [4]. Although erythrocytes are believed to determine the plasma S1P level during the steady state in healthy subjects [5–7], the fact that platelets contain S1P abundantly and release it through activation [8, 9], indicates that S1P derived from activated platelets may play an important role during pathological states in which platelets are activated, such as thrombotic conditions. Atherosclerosis is a chronic inflammatory and thrombotic disease, in which platelets play important roles throughout the various stages of atherogenesis. Recently, the involvement of T cell in this process has been also clarified. T cells are detected even in the early atherosclerotic lesions, their numbers increasing with progression of the lesions, and most of these T cells has been shown to be CD4+ T lymphocytes [10]. Several groups have been working in the characterization of the pathogenic T cell subsets [11]. Moreover, the importance of T cells in atherogenesis has been highlighted by animal studies showing that transfer of CD4+ T cells aggravates [12], whilst CD4+ T cell deficiency attenuates atherosclerosis in apoE^-/-^ mice [13].

In this study, we focused on the effect of S1P on T cell migration, and using Jurkat cells, a human acute lymphoblastic leukemia cell line, as a model, we investigated the involvement of platelet-derived S1P in the cross-talk between platelets and lymphocytes, in comparison with the other lysophospholipids, assuming the pathological thrombogenic condition.

## Results

### Concentration of S1P and other related lipid mediators released from activated platelets

**Figure 1:**
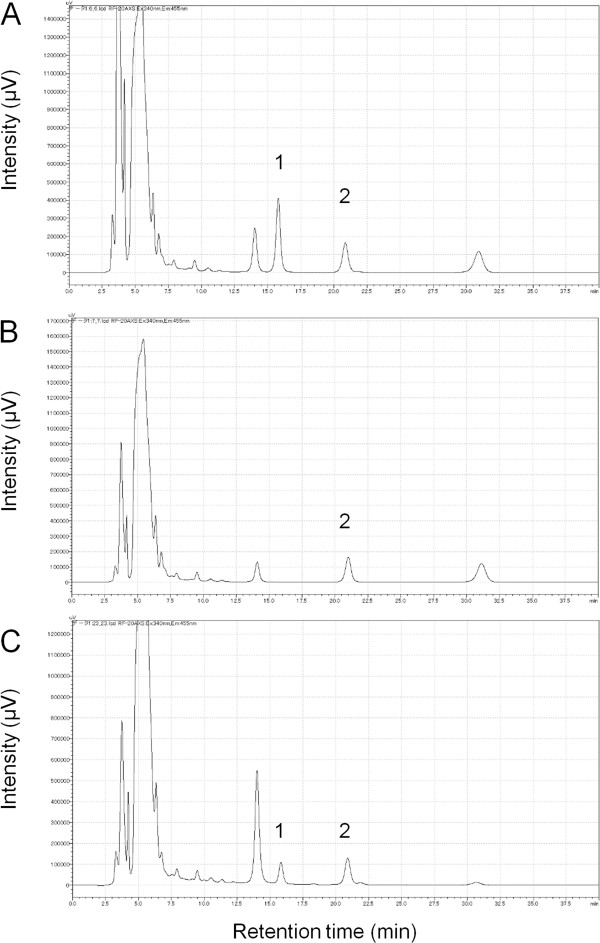
**Measurement of platelet-derived S1P.** Chromatograms of the calibration standard lipids, i.e., C_17_-S1P (internal standard) and S1P **(A)** and absence of C_17_-S1P **(B)**. Chromatogram of the supernatant from activated platelets from a healthy volunteer **(C)**. Peaks 1 and 2 represent C_17_-S1P and S1P, respectively.

We first confirmed lysophospholipids released from platelets. Human washed platelets were stimulated with 20 μg/mL of collagen, and the concentration of S1P in the supernatant was measured. The *ortho*-phthalaldehyde (OPA) derivatives of S1P were separated by HPLC and monitored by fluorescence. The concentration of S1P in the supernatant prepared from activated platelets (5.0 × 10^8^ /mL) was calculated as 0.36 ± 0.11 μM. The chromatograms of calibration standard and platelet derived S1P are shown in Figure 1. The S1P release was found to be collagen concentration-dependent (data not shown). We also analyzed the concentration of other related lipid mediators, namely total phospholipid, LPA and lysophosphatidylcholine (LPC) (Table 1). Autotaxin (ATX), possessing lysophospholipase D activity, produces LPA from LPC, and both LPA and LPC exist in the activated platelet supernatant. The involvement of ATX in the LPA production in this system, however, remains to be solved.

**Table 1:** **The concentration of total phospholipid, S1P, LPA, and LPC from human platelets activated with collagen**

	Concentrations (μM)*
Total phospholipid	11.07 ± 1.96
S1P	0.36 ± 0.11
LPA	0.45 ± 0.21
LPC	4.0 ± 1.41

*mean ± SD (n = 4).

### Expression of S1P receptors and LPA receptors on Jurkat cells

**Figure 2:**
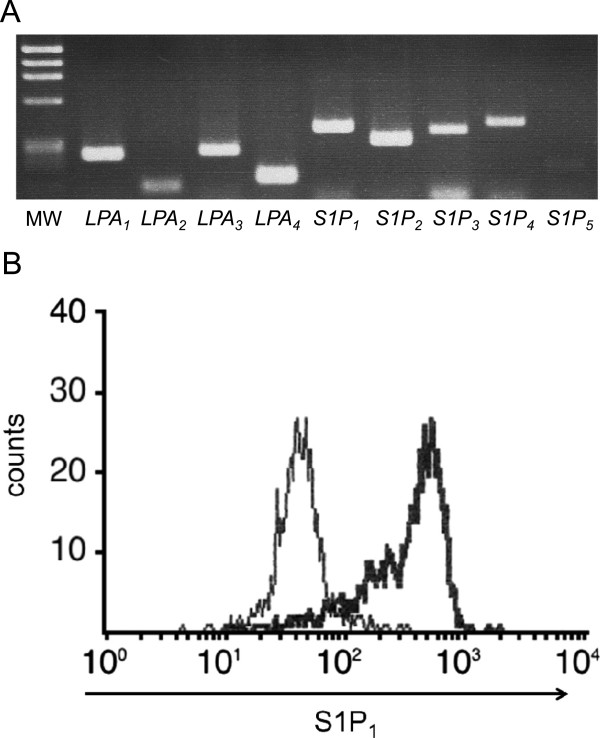
**Expression of S1P and LPA receptors in Jurkat cells. (A)** The expression of S1Ps and LPAs in Jurkat cells was examined by RT-PCR. The amplified products for the Jurkat mRNAs for S1P and LPA receptors were resolved in a 2.5% agarose gel. MW indicates the molecular size markers. mRNA transcripts for S1P_1_, S1P_2_, S1P_3,_ and S1P_4_, were found, but that of S1P_5_ could be hardly detected. Also, LPA_1_, LPA_2_, LPA_3,_ and LPA_4_ were expressed. **(B)** The surface expression of S1P_1_ in Jurkat cells was examined by flow-cytometry. Horizontal axis indicates the strength of S1P_1_ expression as assessed by the indirect FITC fluorescence intensity. The bold line histogram represents staining with anti-S1P_1_ antibody, while the narrow line represents the negative control. S1P_1_ was found expressed on Jurkat cells.

Next, we confirmed the expression of S1P and LPA receptors on Jurkat cells by RT-PCR. As shown in Figure 2-A, Jurkat cells expressed the mRNA transcripts for S1P_1_, S1P_2_, S1P_3,_ and S1P_4_. S1P_5_ mRNA was hardly detectable. In flow-cytometry, we confirmed the expression of S1P_1_ at the protein level (Figure 2-B).

On the other hand, Jurkat cells also expressed LPA_1_, LPA_2_, LPA_3,_ and LPA_4_ (Figure 2-A)_._

### Induction of Jurkat cell migration by S1P and the supernatant of activated human platelets and its reversal by PTX and VPC23019

**Figure 3:**
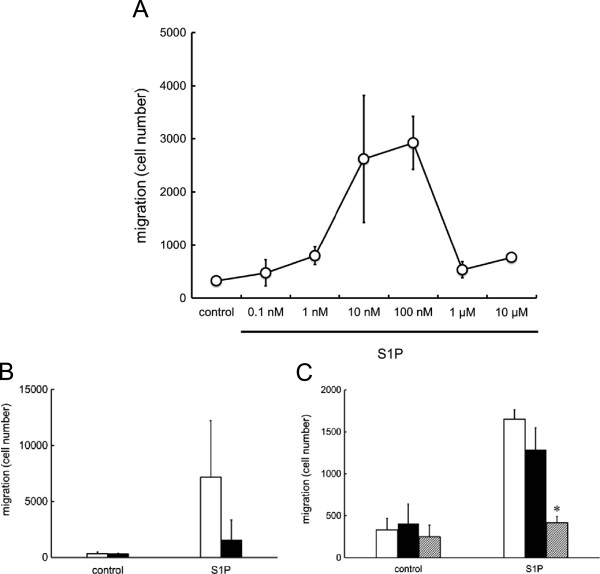
**Jurkat cell migration induced by S1P. (A)** Jurkat cells were seeded into the upper well of the Transwell cell culture chambers, and allowed to migrate to the lower chamber, where various concentrations of S1P were placed, for 4 h. A bell-shape pattern of migration was observed, with a dose-dependent increase of migration with S1P from 0.1 – 100 nM, but decreasing with S1P 1 μM. **(B)** Jurkat cells, pretreated without (open column) or with (solid column) 200 ng/mL of PTX for 60 min, were allowed to migrate toward the indicated concentrations of 100 nM S1P (n = 4). PTX pretreatment inhibited the migration of Jurkat cells induced by S1P. Statistically significant compared to control cells, i.e., without PTX treatment. **(C)** Jurkat cells pretreated without (open column) or with 20 μM (solid column) or with 50 μM (hatched column) of VPC23019 for 60 min were allowed to migrate toward 100 nM S1P (n = 3). VPC23019 at 50 μM, but not 20 μM, significantly inhibited migration of Jurkat cells induced by S1P. *Statistically significant compared to control cells, i.e., without VPC23019 treatment (*P* < 0.01).

**Figure 4:**
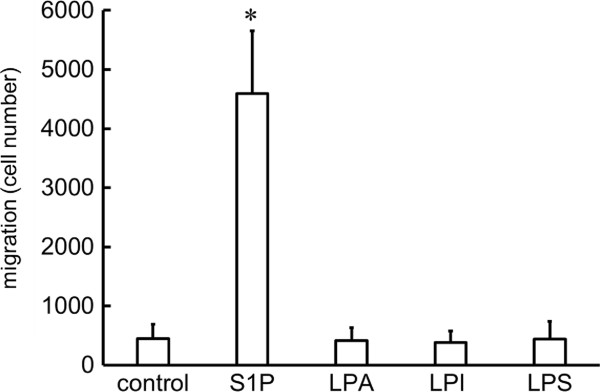
**Jurkat cell migration induced by S1P, LPA, LPI or LPS.** Jurkat cells were seeded into the upper chamber of the Transwell cell culture chambers, and allowed to migrate to the lower chamber, where 100 nM S1P, LPA, LPI or LPS were placed, for 4 h. S1P was the only mediator able to induce migration of Jurkat cells. *Statistically significant compared to cells migrating to well without mediators (*P* < 0.01).

First, the migration of Jurkat cell was evaluated by a modified Boyden’s chamber assay, and the concentrations of S1P leading cells to migrate were confirmed. A marked migration of Jurkat cells was observed with S1P concentration from 10 nM to 100 nM (Figure 3-A), and the migration induced by 100 nM S1P was inhibited by the Gi inactivator PTX (Figure 3-B), and by the VPC23019, in a concentration-dependent manner, with approximately 25% inhibition at 20 μM, and 75% at 50 μM (Figure 3-C). We also evaluated the migration of Jurkat cells in the presence of lysophospholipids other than S1P. In contrast with S1P, LPA, LPI, and LPS did not induce the migration of Jurkat cells (Figure 4).

**Figure 5:**
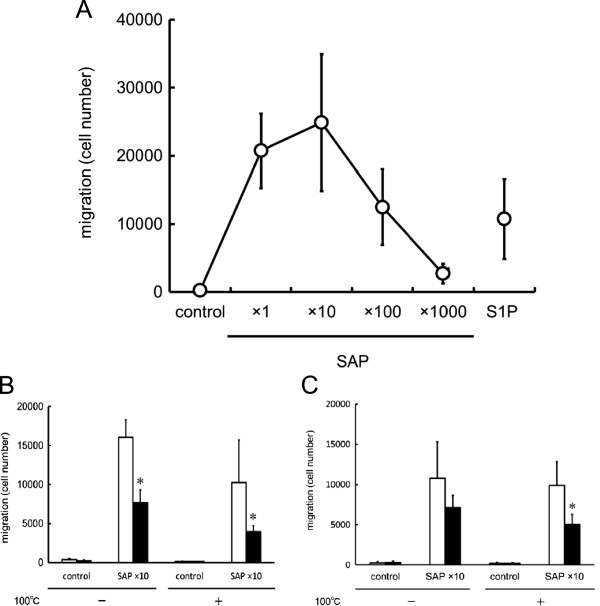
**Jurkat cell migration induced by the supernatant from activated platelets (SAP). (A)** Jurkat cells were seeded into the upper chamber of the Transwell cell culture chambers, and allowed to migrate for 4 h to the lower chamber, where the supernatant from activated platelets, at various concentrations, were placed. A bell-shape migration pattern was observed, with a dose-dependent increase of migration with the supernatant at 1-fold to 10-fold dilution, and decreasing thereafter at 100-fold and 1000-fold dilution. **(B)** Jurkat cells pretreated without (open column) or with (solid column) 200 ng/mL of PTX for 60 min were allowed to migrate toward the indicated concentrations of either heat (100°C)-treated or -untreated supernatant of activated platelets (10-fold dilutions), for 4 h (n = 3). Both heat-treated and –untreated supernatants induced the migration of Jurkat cells, which was inhibited by PTX pretreatment. *Statistically significant compared without PTX treatment (*P* < 0.01). **(C)** Jurkat cells pretreated without (open column) or with (solid column) 50 μM of VPC23019 for 60 min were allowed to migrate toward either heat (100°C)-treated or –untreated supernatant of activated platelets (10-fold dilutions) (n = 3). VPC23019 inhibited the migration of Jurkat cells induced by the supernatant of activated platelets, either heat-treated or –untreated. *Statistically significant compared to VPC23019-untreated cells (*P* < 0.01).

Next, similar experiments were performed using the supernatant of collagen-activated human platelets at a 10-fold dilution. The supernatant induced a strong migration of the Jurkat cells (Figure 5-A), which was also observed when the supernatant was boiled, indicating that the agent responsible for this response was a lipid, but not peptide, mediator (Figure 5-B & C). The migration induced by the supernatant of activated human platelets was inhibited, although not completely, by PTX (Figure 5-B) and VPC23019 at 50 μM (Figure 5-C).

### Induction of Jurkat cell migration by human serum and its reversal by VPC23019

**Figure 6:**
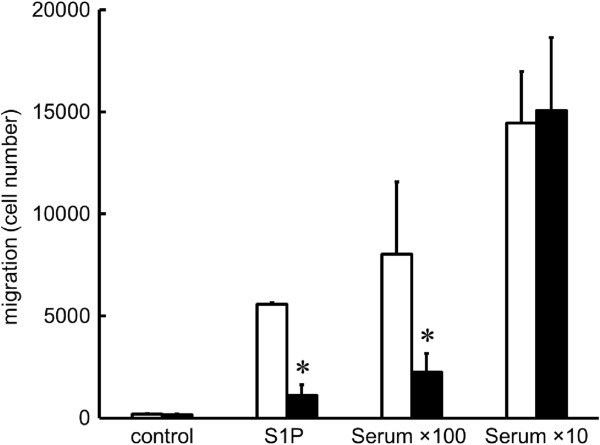
**Induction of Jurkat cell migration by human serum and its reversal by VPC23019.** Jurkat cells pretreated without (open column) or with (solid column) 50 μM of VPC23019 for 60 min were allowed to migrate toward the indicated concentrations of human serum (100-fold dilutions and 10-fold dilutions) (n = 3). Human serum induced strong and dose-dependent migration of Jurkat cells, and the migration induced by human serum at a 100-fold dilution was comparable to that induced by S1P at 100 nM. VPC23019 significantly inhibited the migratory response induced by S1P and human serum at 100-fold dilution, but not that induced by human serum at 10-fold dilution. *Statistically significant compared to cells without VPC23019 pretreatment (*P* < 0.01).

Finally, we evaluated the migratory response of Jurkat cells induced by human serum, into which platelets contents (including S1P) are fully released. Serum-induced Jurkat cell migration was observed; the highest response was observed with 10-fold diluted serum, followed by 100-fold diluted serum. The migratory response induced by 100-fold diluted serum was inhibited by VPC23019, as was the case with S1P (Figure 6).

## Discussion

In this study, among the various effects of S1P, we particularly focused on the cell migration responses, and confirmed the induction of the migratory ability of Jurkat cells, used as the model of T lymphocytes, by S1P released from activated platelets.

In the supernatant of collagen-activated human platelets (5.0 × 10^8^ /mL), the concentrations of S1P and LPA were found to be 0.36 ± 0.11 μM, and 0.45 ± 0.21 μM, respectively. It is reported that 141 ± 4 pmol S1P is stored in 10^8^ platelets [8], and hence mathematically, 51% of S1P stored in platelets was released extracellularly. This figure is reasonable since collagen is a strong platelet agonist. Further, it is reported that human plasma contains about 190 nM S1P, while about 480 nM is present in serum [8], and this difference may be dependent on the activation of platelets.

Under the patho-physiological conditions, such as atherosclerosis, which are associated with thrombosis and platelets activation, the release of lipid mediators is expected to occur. As our results indicate, the other cell types, such as lymphocytes, endothelial cells, macrophages, and smooth muscle cells, which are also involved in the atherosclerotic process, are exposed to S1P released by activated platelets, and are stimulated to migrate. S1P and LPA act mainly through a G protein-coupled cell surface receptors, and as we have confirmed, these receptors are expressed on the Jurkat cells at the mRNA level. S1P_1_ expression was also confirmed on Jurkat T cells by flow-cytometry. We observed that S1P, but not LPA, induced the migration of Jurkat cells, in the modified Boyden’s chamber assay. The migratory ability of Jurkat cells induced by S1P was inhibited by the S1P_1_ (and S1P_3_) antagonist VPC23019 and by the Gi inactivator PTX, as previously reported, confirming the involvement of the S1P_1_ and the Gi signaling pathway. Similarly, the supernatant of activated human platelets also induced the migration of Jurkat cells. This response was preserved either after the heat-treatment of the supernatant, confirming that the mediators released from activated platelets and responsible for the migration response are lipid, but not protein, components. Further, when the concentration S1P in the supernatant prepared from activated platelets was calculated (extrapolated), it corresponded to the concentration of S1P that induced cell migration, and this response was also inhibited by VPC23019 and PTX. It has been reported that S1P_1_, S1P_2_, S1P_3_, and S1P_4_ are expressed on Jurkat cells, and S1P_1_ and S1P_4_ are the mainly expressed on human T lymphocytes [14]. From our experiment using VPC23019, although the involvement of other receptors cannot be completely denied, we considered that S1P_1_ is the mostly involved in the migratory response of T lymphocytes induced by the S1P present in the supernatant of activated platelets.

Similar results are obtained with serum, into which platelets contents (including S1P) are fully released. Measurements of serum concentration of S1P have been extensively performed in our lab, and from our data, the 100-fold diluted serum is known to contain about 10 nM of S1P. Accordingly, 100-fold diluted serum induced the migratory response of Jurkat cells, which was suppressed by VPC23019. However, this inhibitory effect of VPC23019 could not be obtained when cells were stimulated with 10-fold diluted serum. From these findings, it is suggestive that S1P, although not exclusive, plays a pivotal role in the migratory response induced by human serum.

So far, the antagonistic effects of S1P, either by stimulating or inhibiting the process of arteriosclerosis, have been reported. From our present data, it can be suggested that the S1P, released by platelets in the atherosclerotic lesion or other conditions in which platelets accumulate, induces T cells migration and accumulation, accelerating the pathogenesis of the disease. The clinical implications of T cell recruitment to the atherosclerotic lesion by S1P released from activated platelets remain to be elucidated. In *in vitro* studies, however, aspirin and two structurally unrelated reversible cyclooxygenase inhibitors diclofenac and ibuprofen suppressed S1P release. Moreover, oral aspirin intake reportedly attenuated S1P release from platelets in healthy human volunteers *ex vivo* [15]. Further clinical studies are needed to elucidate the importance of local S1P released from platelets and its effects on T cells *in vivo*.

The relationship between atherosclerosis and T cells has already been reported. T cells infiltration is observed even in the early atherosclerotic lesions, their number increasing with the progression of disease; CD4+ T cells are the most abundantly found in atherosclerotic lesions [10]. Several groups have characterized the pathogenic T cell subsets in the arteriosclerotic lesion [11]. Among CD4+ T cells, Th1 cells are pro-atherogenic, while Treg cells are anti-atherogenic and the role of Th2 and Th17 cells remains unknown [16]. Moreover, the importance of T cells in atherogenesis has been highlighted by animal studies showing that transfer of CD4+ T cells aggravates [12], whilst depletion of CD4+ T cells attenuates atherosclerosis in apoE^-/-^ mice [13]. Meanwhile, S1P suppressed the cell migration induced by the chemokines CXCL4 and CXCL-1 [17]. Our results indicated that the concentration of S1P in activated platelet supernatant was consistent with that of purified S1P required to induce migration of Jurkat cell. These facts strongly suggest the involvement of platelet-derived S1P in the pathogenesis of atherosclerosis and the cell-cell interaction between platelets and T lymphocytes through S1P.

Besides atherosclerosis, there are reports on the pathophysiological interaction between platelets and T lymphocytes (lymphocytes). Interaction of platelets with T cells and B cells can contribute to vasculopathy in transplants [18]. Patients with systemic lupus erythematosus (SLE), a condition associated with abnormality of lymphocytes, have been shown to have higher levels of platelet microparticles, CD62P expression, and annexin V compared to control [19]. Platelet-lymphocyte interaction also plays a role in rheumatoid arthritis [20], inflammation in obesity [21], and reperfusion-induced inflammation after cardio-pulmonary bypass (CPB) [22]. Thus, there is the possibility that S1P released by activated platelets induces the platelet-lymphocyte interaction, which is involved in the pathogenesis of arteriosclerosis as well as other clinical conditions, but further research is necessary to confirm these facts.

As referred to in the INTRODUCTION, the S1P_1_ modulator FTY720 has been approved for the treatment of multiple sclerosis, and it has been reported that this agent exerts atheroprotective effects in mice model. Oral FTY720 treatment reportedly results in inhibition of atherosclerosis development in mice via inhibition of effector T responses and induction of a regulatory T-cell response [23]. FTY720 inhibits atherosclerosis by suppressing the machinery involved in monocyte/macrophage emigration to atherosclerotic lesions [24], and by modulating lymphocyte and macrophage function [25]. These findings suggest that FTY720 may represent a new therapeutic approach to atherosclerosis, which is consistent with our present study.

In addition to being an important regulator of lymphocytes in the circulation, our results suggest the important role of S1P for recruiting T lymphocytes at the site of thrombosis where platelets are activated and S1P is accumulated.

## Materials and methods

### Reagents (Materials)

Sphingosine 1-phosphate, and D-erythro (S1P) were purchased from Enzo Life Sciences (Plymouth Meeting, PA). C_17_-sphingosine 1-phosphate (C_17_-S1P), lysophosphatidic acid (LPA), lysophosphatidylserine (LPS), lysophosphatidylinositol (LPI), and VPC23019 were purchased from Avanti Polar Lipids Inc (Alabaster, AL). Pertussis toxin (PTX) was obtained from Wako Pure Chemical Industries (Osaka, Japan).

S1P, LPA, LPS and LPI were dissolved in methanol. Just before use, the methanol was evaporated and the reagents were resolved in PBS containing 0.4% fatty acid-free BSA (Sigma-Aldrich Co., St. Louis, MO). C_17_-S1P was dissolved in methanol. VPC23019 and PTX were dissolved in DMSO.

### Cell culture

The human T-cell leukemia Jurkat cells were maintained in RPMI 1640 (Nacalaitesque, Inc. Kyoto, Japan) with 10% fetal bovine serum (Gibco, NY), 1% antibiotics/antimycotics at 37°C under an atmosphere of 5% CO_2_ and 95% room air.

### Platelet sample preparation

Platelets were isolated from the venous blood of healthy adult volunteers, who had agreed to participate and had given informed consent. Human washed platelets (at a cell density of 5.0 × 10^8^/mL) were prepared as previously described [26], and stimulated with 20 μg/ml of type I collagen (collagen reagent Horm; Nycomed, Munich, Germany) for 15 min under continuous stirring at 1,000 r.p.m. The samples were then centrifuged at 10,000 r.p.m for 1 min, and the resultant supernatants were used to stimulate Jurkat T cells.

### Measurement of S1P

The contents of S1P in the supernatant of platelets were determined using two-step lipid extraction followed by HPLC separation, as described previously [27]. Briefly, samples were sonicated in 3 mL of methanol/chloroform (2:1) with internal standard for 30 min. After adding 2 mL of chloroform, 2.1 mL of 1 mM KCl, and 100 μL of 3N NaOH, samples were centrifuged and the alkaline upper phase (3.8 mL) was collected to new tubes, to which 4 mL of chloroform and 200 μL of concentrated HCl were added. The resultant lower chloroform phases (3.5 mL) formed under nitrogen gas and resolved in methanol, followed by HPLC separation with TSKgel ODS-80TM column (Tosoh, Tokyo, Japan). For the measurement of S1P content, we used C17-S1P as an internal standard.

### Quantitation of related lipids

Choline-containing phospholipids and LPC in the sample were measured using commercially available enzymatic assay kits: AZWELL LPC Assay Kit (Alfresa Pharma, Osaka, Japan) and Nescauto PL-V2 (Alfresa Pharma, Osaka, Japan), respectively. These phospholipid measurements were performed using a Hitachi 7600 analyzer (Hitachi, Tokyo, Japan). The concentration of LPA was determined by an enzymatic cycling assay, as described previously [28].

### RNA isolation and RT-PCR

Total RNA was prepared from Jurkat cells with the total RNA isolation system, ISOGEN (Nippon Gene, Tokyo, Japan). The total RNA (1 μg) was then reverse transcribed using a Transcriptor First Strand cDNA Synthesis Kit (Roche Diagnostics, Tokyo, Japan). The reverse-transcribed complementary DNA was then amplified in a GeneAmp PCR system 9700 (Applied Biosystems, CA, USA). Oligonucleotide primer pairs used for S1P_1_, S1P_2_, S1P_3_, S1P_4_, S1P_5_, LPA_1_, LPA_2_, LPA_3_, LPA_4_. The primer sequences were shown in details in Table 2. The amplification was conducted with 35 cycles of 30 sec at 95°C, 30 sec at 55°C and 30 sec at 72°C (S1P_1_, S1P_2_), 35 cycles of 30 sec at 95°C, 30 sec at 60°C and 30 sec at 72°C (S1P_3_, S1P_4_, S1P_5_, LPA_2_), 40 cycles of 30 sec at 95°C, 30 sec at 55°C and 30 sec at 72°C (LPA_1_, LPA_3_, LPA_4_). The PCR products were resolved by electrophoresis on a 2.5% agarose gel, and then stained with ethidium bromide.

**Table 2:** **List of primers**

Gene	Gene ID	Forward sequence (5′-3′)	Reverse sequence (5′-3′)	Amplicon size (bp)
*S1P*_*1*_	NM001400	CCTCTTCCTGCTAATCAGCG	ACAGGTCTTCACCTTGCAGC	385
*S1P*_*2*_	NM004230	CATTGCCAAGGTCAAGCTGT	ACGATGGTGACCGTCTTGAG	315
*S1P*_*3*_	NM005226	TCAGCCTGTCTCCCACGGTC	ACGGCTGCTGGACTTCACCA	371
*S1P*_*4*_	FJ200495	ACGGGAGGGCCTGCTCTTCA	AAGGCCAGCAGGATCATCAG	420
*S1P*_*5*_	XM005259937	AACCCCATCATCTACACGCTCA	AGCCGCTGAAGCTCCCATCAA	184
*LPA*_*1*_	NM001401	CACATCTTTGGCTATGTTCG	GGGTTCATGGCAGAGTTG	245
*LPA*_*2*_	NM004720	CGCTCAGCCTGGTCAAGACT	TTGCAGGACTCACAGCCTAAAC	109
*LPA*_*3*_	NM012152	CTACGTGTACGTCAAGAGG	ACCCCATCATCTACTCCTAC	259
*LPA*_*4*_	NM005296	AAAGATCATGTACCCAATGACCTT	CTTAAACAGGGACTCCATTCTGAT	139

### Flow cytometry (S1P1 expression)

To examine the S1P_1_ expression of Jurkat cells, cultured cells were collected and incubated with anti-S1P_1_ polyclonal antibody [S1P_1_: Anti-S1P Receptor1 (EDG-1) rabbit polyclonal antibody] (Biomol, Plymouth Meeting, PA, USA). After incubation, the cells were treated with FITC labeled goat anti-rabbit IgG as the second antibody. The number of cells showing positive expression of S1P_1_ was analyzed by FACS flow cytometry (Becton–Dickinson).

### Migration assay

Jurkat cell migration was assessed by means of a modified Boyden’s chamber assay, *i.e.*, in Transwell cell culture chambers (Costar, Cambridge, MA). Polycarbonate filters with 8 μm pores, used to separate the upper and lower chambers, were coated with collagen Type I solution (Cellmatrix Type I-C, Nitta Gelatin Inc, Osaka, Japan). The coated filters were washed twice with a PBS and dried immediately. Then Jurkat cells were added to the upper compartment of the chamber at a density of 1.0 × 10^7^/mL (1.0 × 10^6^ per 100 μL) of medium containing 0.1% fatty acid free bovine serum albumin and incubated for 4 h at 37°C. The Jurkat cells were allowed to migrate toward an indicated reagent (S1P and supernatant of activated platelets; 600 μL) in the lower chamber. After the reaction, migrated cells were collected from the bottom chamber, washed, resuspended in 250 μL and counted by flow cytometry for 1 min. Each determination represents the average of two individual migration chambers. When indicated, cells were preincubated with 20 μM, 50 μM VPC23019 or 200 ng/mL PTX for 60 min.

### Statistics

The results were expressed as the mean ± SD. Where indicated, the statistical significance of the differences between two groups was determined by a paired Student’s *t* test. *P* < 0.01 was considered to denote significance.

## Abbreviations

ATX: Autotaxin
HPLC: High-performance liquid chromatography
LPA: Lysophosphatidic acid
LPC: Lysophosphatidylcholine
LPE: Lysophosphatidylethanolamine
LPI: Lysophosphatidylinositol
LPS: Lysophosphatidylserine
OPA: *Ortho*-phthalaldehyde
PTX: Pertussis toxin
SAP: The supernatant from activated platelets
S1P: Sphingosine 1-phosphate.
